# Developing Consensus Indicators to Assess Pharmacy Service Quality at Primary Health Centres in Yogyakarta, Indonesia

**DOI:** 10.21315/mjms2019.26.4.13

**Published:** 2019-08-29

**Authors:** Satibi Satibi, M Rifqi Rokhman, Hardika Aditama

**Affiliations:** Department of Pharmaceutics, Faculty of Pharmacy, Universitas Gadjah Mada, Yogyakarta, Indonesia

**Keywords:** indicators, modified Delphi method, pharmacy service quality, primary health centre

## Abstract

**Background:**

There have been no existing performance indicators to measure the overall quality of pharmacy services, including the aspects of drug management and clinical pharmacy services, at primary health centres in Indonesia. This study aimed to obtain these indicators based on a consensus of experts.

**Methods:**

The modified Delphi method was used to obtain the consensus. The initial indicators, based on a literature review, were evaluated and assessed by members of the expert panel through three rounds of repetition until the consensus was reached. The expert panel members were selected based on their knowledge of or expertise in pharmacy service performance and geographical considerations. Analysis of the expert panel consensus level was determined by calculating the mean and interquartile range.

**Results:**

Fifteen expert panel members started the first round (93.7% of the 16 targets) with 12 of them (75%) completing the third round of the modified Delphi method. Three expert panel members were representatives of the Regency Health Office, and the others were pharmacist practitioners at primary health centres from three different regencies. The consensus results were 26 indicators of drug management, 19 indicators of clinical pharmacy services, and two indicators of overall pharmacy performance.

**Conclusion:**

The consensus indicators for measuring drug management, clinical pharmacy services, and overall pharmacy performance can be used as a reference and standard to measure the quality of pharmacy services at primary health centres. Therefore, the measurement results are more relevant if compared between one and other studies.

## Introduction

Universal health coverage has been acknowledged as a key health target in the Sustainable Development Goals and has become a major goal for health reform in many countries (1, 2). The World Health Organization stated that the goal of this health care reform is “to ensure that all people obtain the health services they need without suffering financial hardship when paying for them” (3). The Indonesian government started a National Health Insurance programme beginning 1 January 2014, where all residents who are members of this health insurance scheme can visit an appointed primary health centre without a prior appointment. A primary health centre provides health care services from a non-specialist health care worker who is accessible on a first point of contact basis (4). Hence, the primary health centre is highly strategic as it is provided in every district to facilitate access to health services for all residents (5). The patients need a referral letter from a primary health centre if they seek further treatment in hospitals or specialist clinics (4).

A new regulation in 2016 set the standard for the implementation of pharmacy services in primary health centres (6). This regulation defines standards for (i) management of drugs and disposable medical supplies and (ii) clinical pharmacy services. Even though these minimum standards have been determined, in practice, many primary health centres have not met these standards; for instance, there is poor drug management at several primary health centres (7, 8).

Although indicators are required to assess and improve the quality of pharmacy services, there was no existing indicator for measuring the overall quality of pharmacy services at primary health centres in Indonesia in terms of drug management and clinical pharmacy services. Some studies from other countries have developed clinical pharmacy key performance indicators and have even documented clinical pharmacy services using personal digital assistants (9, 10). However, performance indicators developed in other countries cannot be directly applied in Indonesia since there are many differences in the health care systems.

Indicators that have been widely studied in Indonesia have focused mainly on drug management and have not included indicators to measure aspects of clinical pharmacy services (7, 11, 12). The indicators used for measuring drug management also vary across studies; for instance, dead stock was measured in one study (11) but was not measured in other studies (7, 12, 13). In addition, some studies measured patient satisfaction as the sole indicator of pharmacy service (14). The differences in the indicators used made the research findings and pharmacy service performances at the observed primary health centres less relevant for comparison. Therefore, a consensus is needed from an expert panel to compile appropriate indicators.

The Delphi method is a technique used to reach consensus within a group (15). This method can systematically collect and combine assessments through several repetitions, based on information from a group of experts. The main characteristics of the Delphi method are that members of the expert panel are anonymous, which allows for changing views from a previously held position without embarrassment; controlled feedback is regulated by a non-voting chairman, which prevents the process from being hijacked by a vocal minority; and there is repetition of an assessment until a consensus is reached (16–18). Feedback from successive rounds encourages the expert panel to re-assess, change, and/or develop their opinions (19, 20). This method is appropriate when there is an absence of agreement, a lack of information for decision-making or uncertainty or lack of conclusive evidence (21).

Drug management indicators are needed to support drug availability and efficient drug inventory management, while clinical pharmacy service indicators are needed to prevent the occurrence of drug-related problems, which ultimately aims to improve patient safety. Assessment of the quality of pharmacy services for a primary health centre must incorporate an assessment of both aspects. Therefore, this study proposed to determine the indicators along with the method of assessing these indicators based on an expert panel consensus using a modified Delphi method to measure the quality of pharmacy services in primary health centres in terms of both drug management and clinical pharmacy services.

## Methods

Consensus indicators of pharmacy service quality were obtained through the modified Delphi method. The Delphi method is widely used and accepted as a method for collecting data from experts within their area of expertise (19). In this modified method, there were two groups: (i) working groups composed of researchers who compiled the initial indicator instruments based on a review of the existing literature and (ii) consensus groups, who were members of the panel of experts who assessed and provided input on the quality indicators of pharmacy services at the primary health centres. At the time of discussion, the panel members were led by the nonvoting chairman. The arrangement of this group was adjusted to match previous studies that used the modified Delphi method (18).

In summary, the main processes in this method included the development of initial indicator instruments by working groups based on a literature review, the selection of expert panel members, the repetition of expert panel assessments, and a consensus assessment using the modified Delphi method.

## Initial Indicator Instrument

Round 1 of the traditional Delphi method started with an open-ended questionnaire. The answers to open questions would then be used by researchers to compile another questionnaire that was assessed by an expert panel in the next round. However, modifications could be made to the Delphi method whereby Round 1 started with questionnaires designed by the working group based on a literature review (19, 22). In this study, the modified Delphi method was used, and the modification was carried out on an initial indicator instrument developed based on the existing literature review.

The initial instrument was a questionnaire containing a list of pharmacy service quality indicators divided into indicators for drug management and indicators for clinical pharmacy services based on the standard of pharmacy services at primary health centres in Indonesia (6). The working group developed drug management performance indicators that included indicators for selection of drugs, planning, requesting and receiving, storing, distributing, controlling, recording and reporting, and evaluation of drug management (6, 23, 24). Clinical pharmacy service indicators included assessing and prescribing services, drug information services, counseling, patient visits (specifically for the primary health centres that serve inpatients), monitoring of drug side effects, monitoring of drug therapy, and evaluation of drug use (6). Finally, patient satisfaction was added as an overall performance indicator because it was used in several previous studies.

## Selection of Expert Panel Members

The modified Delphi method is very dependent on expert dynamics. The selection of expert panels for this study took into account two primary factors, as follows: (i) Panel members should have demonstrated knowledge or expertise in pharmacy services at primary health centres. For this reason, the people targeted for the expert panel included the chairperson of the Provincial Health Service Quality Agency, representatives from the Regency Health Office and primary health centre pharmacist practitioners. The Health Service Quality Agency is responsible for fostering primary health centres in meeting quality standards and patient safety in accordance with applicable accreditation and regulatory standards. Representatives from the health officials were selected by appointing the persons in charge of managing the regency pharmacy department, which supplies the drugs needed at primary health centres. The expert panel members from primary health centres must be pharmacists because of the regulation stating that the pharmacy at primary health centres must be managed by a pharmacist (6). (ii) In addition, geographical considerations were taken into account by involving representatives of expert panels from the three regencies of Yogyakarta City, Sleman Regency and Bantul Regency. These three regencies were selected because all pharmacies at public health centres in this area are supervised by a pharmacist. Because each regency has the autonomy to regulate primary health centres, having representatives from several agencies led to a diversity of expert panel members. That, in turn, led to better performance because it allowed for the consideration of different perspectives (25).

## Delphi Rounds

In theory, the Delphi method allows for continuous repetition until a consensus is reached among the experts. However, some studies have stated that in many cases, three rounds were enough to gather information and reach consensus (16, 22), so in this study, the three-round modified Delphi method was used.

In between each of the three votes, indicators were revised by the working group based on the feedback from the consensus group. All votes were anonymous. In Round 1, the expert panel provided an assessment of each indicator using a Likert scale and recommended changes by removing, adding and/or altering the diction of the existing indicators. Afterward, they continued with a discussion. The researchers changed the pharmacy service quality assessment indicators based on the results of Round 1 (including changes according to the suggestions made by the experts and the results of the discussion session).

In the original Delphi method, experts never meet or interact directly, which means that information is only exchanged between individuals (who may be numerous and geographically dispersed) in an iterative process. This is done in the belief that there will be benefits from the exchange of information at a low cost. In addition, this exchange is strictly controlled to limit the potentially detrimental effects of interaction (25). However, one criticism about the traditional Delphi method is that it does not facilitate meetings of the members of the expert panel although interaction between the experts is highly significant in a complex decision-making process requiring clarification of the language used and recommendations to be made (15). Therefore, this study employed a modified Delphi method that allowed for meetings of the experts in discussion sessions. To reduce bias due to the interaction of experts, the discussion was more directed at equating the perceptions of expert panel member rather than changing the assessment of the expert panel.

In Round 2, the expert panel provided a reassessment of each revised indicator from Round 1 and recommended changes by removing, adding and/or altering the diction. In Round 2, the expert panels could change their assessments and after that, a discussion session was held. The researchers changed the pharmacy service quality assessment indicators based on the results of Round 2 (including changes according to feedback from the expert panel). Finally, Round 3 involved the expert panels providing a final reassessment of indicators that had been revised after Round 2.

## Data Analysis for Consensus Assessment

A Likert scale from 1 (strongly disagree) to 7 (strongly agree) was used to assess the approval of expert panel members on indicators. Consensus measurement is an important component of Delphi analyses and data interpretation (17). But there is no agreement on the level of approval that is the best approach in the modified Delphi method (26), so in this study, two indicators were selected for consensus assessment: the mean and interquartile range (IQR). This study adopted the approach of several previous studies, where consensus was obtained if the indicator had a minimum mean value of 70% or a value of more than 4.9 (out of a maximum value of 7) (26, 27) and where an IQR of 0–1 meant a high level of consensus, 1.01–1.99 meant moderate consensus and more than 2 meant no consensus (28). For Round 3, a consensus on indicators was reached if the mean value was above 4.9 and the IQR scores were in the moderate or high level of consensus category.

## Results

The target number of expert panel members was 16 and all of them were willing to take part in the research. However, only 15 members of the panels participated in Round 1 (93.7% of the target), 15 (93.7%) participated in expert panels in Round 2 and 12 (75%) participated in the expert panels in Round 3 (Table 1). Those who did not take part in Round 1 were representatives of the Health Service Quality Agency and in Round 3, there were three experts who were not present because the primary health centres where they worked were being accredited. Of the 15 experts, three pharmacists (20%) came from the Regency Health Office, while the other 12 (80%) were pharmacist practitioners at primary health centres. The mean age was 35.7 years (range 25–44 years), and the average length of practice was 9.4 years (range 3–16 years). A description of characteristics of the expert panel can be seen in Table 2.

Discussion sessions were held between Round 1 and Round 2 and between Round 2 and Round 3. According to feedback from the expert panel during Round 1 and the first discussion session, there were two indicators omitted because they were not relevant to the current state of pharmacies at primary health centres (Table 3). Those indicators had low mean values and IQR values of 2.

The expert panels provided recommendations for three indicators, which were included in Round 2, namely, the suitability of drug items received, the accuracy of the numbers of a drug distributed, and documentation of prescription screening. In Round 2, the expert panel also suggested four additional indicators, which were included in Round 3. These were the percentage of unprescribed drug items, or dead stock (> 3 months); drug costs per prescription; patient compliance; and the use of oral rehydration solutions (ORS) and zinc for diarrhea.

Seven indicators were also omitted or combined with other indicators following written recommendations of the panel experts in Round 2 in order to avoid duplicate measurements. These indicators were the suitability of a drug item requested, suitability of a drug item received, accuracy of items distributed, availability of vaccines, values of defective drugs, documentation of prescription screening and documentation of provision of patient counseling. These indicators were not included in Round 3.

Consensus indicators, their assessment methods, and the assessments of the expert panel are shown in Table 4 (26 indicators of drug management), Table 5 (19 indicators of pharmacy services), and Table 6 (two indicators of overall pharmacy performance).

Consensus assessment based on mean values showed that the number of indicators agreed upon by the expert panel increased in each successive round (Table 7). However, for consensus assessment based on IQR, there were still indicators in the moderate consensus category.

For Round 3, there were two indicators that did not have agreement from members of the expert panel, which were drug storage indicators according to pharmacological effects, with a mean value of 5.58 but IQR values of 2.25 (no consensus), and the average length of time for out-of-stock drugs, with an IQR of 2 (no consensus). Therefore, these two indicators were not included in the consensus indicators.

## Discussion

The potential for a low response rate was a crucial issue that had to be addressed in using the Delphi method, given that this method requires a long time to complete and repeated assessments (19). However, this study received a fairly high response rate of 12 out of 16 experts (75%) willing to follow the Delphi method process through Round 3.

Among the panel of experts, there were two expert pharmacists who had worked at a primary health centre for less than 5 years. This occurred because the previous regulation did not mandate that a pharmacist should manage the pharmacy at a primary health centre. In the past, pharmacies at primary health centres were managed by a pharmacy technician. However, the implementation of Government Regulation Number 51 of 2009 regulated that drug inventory management must be managed by a pharmacist (29). In addition, Regulation of Minister of Health Number 30 of 2014 clearly stated that each pharmacy at a public health centre should be managed by a pharmacist and public health centres were given 3 years to recruit a pharmacist (30). Hence, the majority of pharmacists at public health centres have less than 5 years of experience.

Other studies have only included statements (indicators) that reached consensus in the previous round, and statements that did not receive consensus were omitted in the next round. However, this means that statements failing to meet consensus are eliminated without the opportunity for members of the expert panel to change their initial assessment (16). Therefore, in this study, all indicators in a round were adjusted based on the expert panel recommendations and included in the next round.

The traditional Delphi method was criticized in one study for reducing the positive aspects of face-to-face interaction for the exchange of information that would help to identify the reasons for a dispute (25). To avoid this problem, the modified Delphi method used in this study included a meeting session of the expert panel members to provide opinions (15) and equalise their views. This session was led by a nonvoting chairman who was a representative of the researchers.

These meeting sessions provided another benefit by reducing the loss of important indicators due to lack of understanding among the panel of experts on these indicators. This information and feedback process permitted and encouraged the selected Delphi participants to reassess their initial judgments about the information provided in previous iterations (19). For example, the inventory turnover ration (ITOR) had a mean value of 3.21 and 4.73 (below the mean value of 4.9) in Rounds 1 and 2, with an IQR of 3 (no consensus) in Round 2. However, ITOR is a commonly used indicator of the effectiveness of inventory management or inventory control (24). During the second discussion session, several expert panel members said they gave it low marks because the terminology used regarding the indicator and how to measure it was unusual. However, after discussion with other expert panel members in Round 3, there was a high level of consensus for this indicator.

It appeared to be easier to reach consensus with analyses based on the mean than to reach consensus with analyses based on IQR (Table 7). In Round 3, the mean values for all indicators had high levels of consensus, while the IQR indicators had reached only moderate consensus. Two indicators that had low consensus in Round 3 were the indicators of drug storage based on pharmacological effects and the average time of drug stock-outs with mean values >4.9, and no consensus was reached based on IQR (IQR value of 2).

For the drug storage indicators, despite the particular storage method based on pharmacological effects being able to minimise the potential for medication errors, it received low consensus because it also required a lot of space and was, therefore, considered difficult to implement at the primary health centres. For indicators of the average time for a drug to be out-of-stock, there was low consensus because the drugs are sent by the regency pharmaceutical installation so that this indicator was more appropriate for assessing the performance of the regency pharmacy installation.

The final slate of indicators of the quality of pharmacy services at primary health centres consists of 26 indicators for drug management, 19 indicators for clinical pharmacy services, and two indicators for overall performance. These indicators not only focus on processes but also on outcomes, as suggested by another study (31). For instance, the consensus indicators of percentage of patients receiving counseling and the use of preventive antibiotics for patients with non-specific diarrhea were expected to be a reference for measuring the quality of pharmacy services so that the use of these indicators could simplify performance comparisons and measurement data interpretation. Formulating indicators according to the stages of drug management would facilitate a follow-up plan for improving pharmacy service performance.

This study has some limitations. One of the problems that arose in using the Delphi method was the potential for competing interests (15). During one of the study’s discussion sessions, a panel expert revealed his disagreement with an existing indicator being used to assess the primary health centre where he practiced because of concerns that it would not have good performance in that area. However, eliminating such a conflict of interest by removing the panel of experts would pose a potential risk of missing people who are knowledgeable about the topic for which consensus was being sought (15). Thus, to reduce the conflict of interest, during that discussion session and in the instructions for Rounds 2 and 3, the researchers reminded panel members to place themselves in the role of expert assessing whether an indicator was needed in general rather than taking the role of the party to be assessed. Despite a conflict of interest being detected during one discussion session, the use of the modified Delphi method with a discussion session presented benefits by reducing the potential for conflicts of interest and creating an equal understanding among the members of the expert panel.

Another limitation to be considered is that two expert panel members had only worked as pharmacists at a primary health centre for 3 years. There is little agreement in the literature about what constitutes an expert, but it is usually defined as a person who has considerable knowledge or experience in the specific field of study (32). However, based on this definition, there is still debate regarding how to define knowledge and experience. Although knowledge and expertise cannot be assumed through the number of years in practice (33), this criterion was used in this study for selecting expert panel members from pharmacists. Before the selection process, the researchers ranked all pharmacists in each regency based on practice experience and invited the top three pharmacists of each regency.

## Conclusion

Fifteen (out of 16 targeted or 93.7%) expert panel members started the first round and 12 (75%) completed the third round of the modified Delphi method. Three expert panel members were representatives of the Regency Health Office, and the others were pharmacist practitioners at primary health centres from three different regencies. The consensus resulted in 26 indicators for drug management, 19 indicators for clinical pharmacy services, and two indicators for overall pharmacy performance. These performance indicators for measuring drug management, clinical pharmacy services, and overall pharmacy performance can be used as a standard reference to measure the quality of pharmacy services at primary health centres, resulting in more uniform and relevant comparisons between studies.

## Figures and Tables

**Table 1:** Distribution of expert panel

Category	Number of persons invited	Agreed to participate	Completed Round 1	Completed Round 2	Completed Round 3
Chairperson of the province health service quality agency	1	1	0	0	0
Representative of the regency health office	3	3	3	3	3
Pharmacist practitioners at primary health centres	12	12	12	12	9

Total	16	16	15	15	12

**Table 2:** Characteristics of expert panel

Category	Information	Number, *n* = 15 *n* (%)
Profession	Regency-level health officer	3 (20.0)
	Pharmacist practitioners at primary health centres	12 (80.0)
Sex	Male	2 (13.3)
	Female	13 (86.7)
Age	20–30 years old	2 (13.3)
	30–40 years old	9 (60.0)
	40–50 years old	4 (26.7)
Region	Yogyakarta city	5 (33.3)
	Sleman Regency	5 (33.3)
	Bantul Regency	5 (33.3)
Practice experience	3–5 years	2 (13.3)
	> 5–10 years	7 (46.7)
	> 10–15 years	4 (26.7)
	> 15–20 years	2 (13.3)

**Table 3:** Indicators omitted for Round 2

Indicator	Mean	IQR	Reason
Frequency of procurement of each drug item per year	2.85	2	At the primary health centres, there was a standard operating procedure in which procurement was carried out for each month so that this indicator was not relevant.
Frequency of incomplete letters of order	3.17	2	To make an order, the primary health centres wrote an order letter directed to the Regency Pharmacy Installation. If the order letter was incomplete, it would not be processed. This event was also very rare.

**Table 4:** Consensus indicators of drug management

No.	Indicator	Round 1		Round 2		Round 3	
A. Drug selection		Mean	IQR	Mean	IQR	Mean	IQR
1	Propose new drugs to be listed or delisted from formulary	5.21	1	6.09	0	6.00	0.5
B. Drug planning							
2	Suitability of drug item with the national formulary	6.36	1	6.42	1	6.42	1
3	Suitability of drug items with disease patterns	3.64*	3*	6.00	1	6.00	1
4	Adequate funds to fulfil out-of-stock drugs	4.93	2*	5.88	0.5	5.33	1
5	Planning accuracy	5.57	0	6.27	0.5	6.25	1
C. Drug order and receipt							
6	Suitability of the number of drug items requested	5.69	0	5.83	1	6.00	1
7	Suitability of the number of drug items received	4.69*	4*	5.92	1	6.33	1
D. Drug storage							
8	Drug storage according to dosage form	6.29	0.75	6.58	1	6.33	1
9	Drug storage according to temperature	6.14	0.75	6.58	1	6.67	1
10	Narcotics storage according to regulations	6.15	1	6.58	1	6.75	0.25
11	Drug storage is not used for storing other items that cause contamination	6.21	1	6.08	1	6.67	1
12	Drug arrangement follows FEFO method	6.00	1	6.50	0.25	6.83	0
13	High-alert drug storage	5.64	0	6.33	1	6.67	1
14	LASA drug storage	5.71	0	6.58	1	6.58	1
15	Storage of drugs removed from the primary packaging	4.50*	2.75*	6.25	1	5.83	1.25
E. Drug distribution							
16	Accuracy of the number of drugs distributed to pharmaceutical service sub-unit	-	-	5.75	1.25	5.83	0.25
F. Drug controls							
17	Inventory turnover ratio (ITOR)	3.21*	0.75	4.73*	3*	5.92	0.5
18	Availability level of drugs (month units)	5.14	0	5.83	0	6.08	0
19	Out-of-stock drug items (< 1 month)	5.36	0.75	6.17	0.25	6.00	0.5
20	Shortage inventory of drug items (1–< 12 months)	4.38*	3*	6.00	0.25	5.92	1.25
21	Adequate inventory of drug items (12–18 months)	4.29*	3.75*	5.75	0	6.08	2
22	Overstock dug items (> 18 months)	4.07*	3.75*	5.45	1	5.83	1
23	Not prescribed drug items or dead stock (> 3 months)	-	-	-	-	5.92	0.25
24	Values of expired and defective drugs	5.23	0	5.75	1.5	6.42	1
G. Recording, reporting and archiving							
25	Accuracy of the physical amount of the drug with the amount on the stock cards or computer	6.00	0	6.25	1	6.42	1
H. Drug monitoring and evaluation							
26	Periodic evaluation of drug management	6.07	0.75	6.17	1	6.00	1.25

Note:

* Indicator has not reached consensus

FEFO: First expired, first out

LASA: Look-alike, sound-alike

**Table 5:** Consensus indicators of clinical pharmacy service

No.	Indicator	Round 1		Round 2		Round 3	
A. Assessing and prescribing		Mean	IQR	Mean	IQR	Mean	IQR
1	Prescription screening	-	-	6.17	1	6.42	1
2	Labeling for dispensed drugs	5.86	0	6.33	1	6.25	1.25
3	Providing drug information when delivering drugs to the patients	6.21	0	6.50	1	6.75	0
4	Service time	6.07	0	6.50	1	6.50	1
5	Polypharmacy	5.50	1.5	6.42	1	6.33	1
B. Drug information services							
6	Documentation of drug information services	6.07	0.75	6.18	1	6.08	1
C. Counseling							
7	Providing patient counseling	3.75*	2.25*	6.27	1	5.92	1.25
D. Ward pharmacist services (only for primary health centres that offer inpatient service)							
8	Documentation of ward pharmacist services	6.09	0.5	6.40	1	6.17	1
E. Side effect monitoring							
9	Documentation of side effect monitoring	6.00	0	6.33	1	6.08	0
F. Monitoring of drug therapy							
10	Documentation of drug therapy monitoring	5.93	0	6.00	0.25	5.67	1
G. Evaluation of drug use							
11	Drug costs per prescription	-	-	-	-	5.92	1.25
12	Items per prescription	6.29	1	5.83	1	6.25	1.25
13	Generic pharmaceutical products	5.93	0.75	6.00	1	5.83	1.25
14	Antibiotics in non-specific diarrhea	6.36	1	6.58	1	6.58	1
15	Giving oral rehydration solutions (ORS) and zinc for diarrhea	-	-	-	-	6.08	1
16	Antibiotics in acute respiratory infections, non-pneumonia	6.36	1	6.58	1	6.58	1
17	Avoiding injection for patients with myalgia	6.07	1	5.92	1	6.33	1
18	Patient compliance	-	-	-	-	5.67	0.25
19	Documentation of medication errors	6.33	1	6.45	1	6.58	1

Note:

* Indicator has not reached consensus

**Table 6:** Consensus indicators of overall pharmacy performance

No	Indicator	Round 1		Round 2		Round 3	
		Mean	IQR	Mean	IQR	Mean	IQR
1	Patient satisfaction	6.21	0	6.25	0.25	6.33	1
2	Continuity of patient satisfaction survey	6.15	0	6.17	0.25	6.17	1.25

**Table 7:** Consensus assessment based on mean and IQR in each round

Indicator	Mean values	Number of indicators			Consensus category based on mean	IQR values	Number of indicators			Consensus category based on IQR
	
		Round 1	Round 2	Round 3			Round 1	Round 2	Round 3	
Drug management	> 4.9	20	31	26	High	< 1	20	26	22	High
	< 4.9	12	1	2	Low	1.01–1.99	0	3	4	Moderate
						> 2	12	3	2	No
Clinical pharmacy service	> 4.9	16	18	19	High	< 1	15	18	14	High
	< 4.9	1	0	0	Low	1.01–1.99	1	0	5	Moderate
						> 2	1	0	0	No
Overall performance	> 4.9	2	2	2	High	< 1	2	2	1	High
	< 4.9	0	0	0	Low	1.01–1.99	0	0	1	Moderate
						> 2	0	0	0	No

Note: In Round 3, two indicators of drug management have not reached consensus
